# Integrating Oral PrEP Into Family Planning Services for Women in Sub-saharan Africa: Findings From a Multi-Country Landscape Analysis

**DOI:** 10.3389/frph.2021.667823

**Published:** 2021-06-18

**Authors:** Neeraja Bhavaraju, Rose Wilcher, Regeru Njoroge Regeru, Saiqa Mullick, Imelda Mahaka, Jessica Rodrigues, Jennifer Mason, Jane Schueller, Kristine Torjesen

**Affiliations:** ^1^Afton Bloom, Washington, DC, United States; ^2^Global Health, Population and Nutrition, FHI 360, Durham, NC, United States; ^3^LVCT Health, Nairobi, Kenya; ^4^Wits Reproductive Health and HIV Institute, Faculty of Health Science, University of the Witwatersrand, Johannesburg, South Africa; ^5^Pangaea Zimbabwe AIDS Trust, Harare, Zimbabwe; ^6^AVAC, New York, NY, United States; ^7^Office of Population and Reproductive Health, United States Agency for International Development, Washington, DC, United States; ^8^Office of HIV/AIDS, United States Agency for International Development, Washington, DC, United States

**Keywords:** HIV prevention, PrEP, PrEP-FP integration, SRH-HIV integration, AGYW

## Abstract

Integration of HIV and family planning (FP) services is a renewed focus area for national policymakers, donors, and implementers in sub-Saharan Africa as a result of high HIV incidence among general-population women, especially adolescent girls and young women (AGYW), and the perception that integrating HIV pre-exposure prophylaxis (PrEP) into FP services may be an effective way to provide comprehensive HIV and FP services to this population. We conducted a focused desk review to develop a PrEP-FP integration framework across five key categories: plans and policies, resource management, service delivery, PrEP use, and monitoring and reporting. The framework was refined via interviews with 30 stakeholders across seven countries at varying stages of oral PrEP rollout: Kenya, Lesotho, Malawi, South Africa, Uganda, Zambia, and Zimbabwe. After refining the framework, we developed a PrEP-FP integration matrix and assessed country-specific progress to identify common enablers of and barriers to PrEP-FP integration. None of the countries included in our analysis had made substantial progress toward integrated PrEP-FP service delivery. Although the countries made progress in one or two categories, integration was often impeded by lack of advancement in other areas. Our framework offers policymakers, program implementers, and health care providers a road map for strategically assessing and monitoring progress toward PrEP-FP integration in their contexts.

## Introduction

The region of East and southern Africa is the most affected by HIV, with over 700,000 new infections in 2019 (1). Women are disproportionately affected, as demonstrated by 2018 HIV prevalence rates among young women (15–24 years), which are more than double the rates seen among young men (1). Oral pre-exposure prophylaxis (PrEP) and the future introduction of new biomedical products—such as the dapivirine vaginal ring, long-acting injectable cabotegravir, and multipurpose HIV prevention and contraceptive technologies—have the potential to substantially reduce new HIV infections if these products can be accessed and effectively used by those at risk of HIV (2–4).

However, the rollout of oral PrEP has been confronted with many challenges, including difficulties translating policy into practice and optimizing access, uptake, and effective use among populations at risk of acquiring HIV in sub-Saharan Africa (5). PrEP uptake and use among adolescent girls and young women (AGYW), in particular, have been impeded by barriers including low perceived HIV risk, pill burden, limited private storage space, fear of inadvertent disclosure to family and partners, intimate partner violence, stigma associated with an antiretroviral-based product, and negative attitudes among healthcare providers toward adolescent sexuality and PrEP use (6–8). In addition, although some decentralized, community-based models of PrEP delivery are emerging, PrEP services have largely been provided through specialized HIV or STI clinics, where AGYW do not routinely seek care and that primarily target key populations, such as female sex workers, men who have sex with men, and transgender women (9).

A potential solution for increasing access to and uptake of oral PrEP among women is to integrate oral PrEP counseling and delivery in family planning (FP) services, which are well-established and well-utilized by sexually active women in many settings. More than one-quarter (28.5%) of women in sub-Saharan Africa of reproductive age (15–49 years) use a modern contraceptive method, including 24.7% of AGYW (15–24 years) (10). Contraceptive prevalence among AGYW is even higher in high HIV burden countries such as Lesotho (59.2%), Zimbabwe (50.7%), and Kenya (36.8%) (10). Half of modern contraceptive users use short-acting methods, such as the daily pill or 3-month injection, both of which typically require the same clinic visit schedule as oral PrEP (11). As a result, integration of FP and oral PrEP services have potential for alignment. Service integration has also received increased attention following the results of the Evidence for Contraceptive Options and HIV Outcomes (ECHO) trial, which found high HIV incidence among women accessing contraceptive services in three high HIV burden countries, with HIV risk greatest among women younger than 24 years (12). These results prompted the World Health Organization and other stakeholders to endorse providing HIV prevention options, including PrEP, in FP services in high HIV burden settings, asserting that scale-up of oral PrEP and other future HIV prevention products in these settings may more effectively reach priority populations such as AGYW where they already receive preventive health services (13, 14).

The attention being given to integrating PrEP into FP services (referred to as PrEP-FP integration henceforth) builds on more than two decades of advocacy, programmatic efforts, and research aimed at strengthening the integration of sexual and reproductive health (SRH) and HIV services more broadly. These efforts, which embrace a woman-centered, choice-based, and rights-based approach to service delivery, have introduced a range of service integration models for different combinations of services, all with the goal of achieving better SRH and HIV outcomes. Although studies suggest that clients have a preference for integrated SRH-HIV services, the evidence of the effects of integrated services on service quality and client outcomes is mixed (15–19). Moreover, these efforts have suggested that achieving service integration requires overcoming challenges to integration throughout the health system, including in policies and guidelines, financing mechanisms, demand creation, monitoring and evaluation, supply chains, and human resource capacity (16). However, the non-integration of some of these health system “hardware elements” (primarily related to structure and resources) may be mitigated by strong “software elements,” such as leadership, management, and provider motivation, agency, and relationships, all of which are essential enablers of effective SRH-HIV integration (20).

The evidence base pertaining to PrEP-FP integration specifically is limited, in part because PrEP is relatively new to the market, with the first African regulatory approvals in 2015. Early demonstration projects suggest that PrEP delivery in FP settings is feasible; however, uptake of PrEP by screened and eligible AGYW in these studies was low, ranging from 4 to 16% (21, 22).

To unlock the potential to meet the HIV prevention needs of women by integrating PrEP delivery into FP services, programs need practical guidance on how to overcome challenges to PrEP-FP integration throughout the health system and achieve sustainable integration of these two services at scale. Drawing on an existing product introduction framework developed to support national rollout and scale-up of oral PrEP (23), a desk review of relevant SRH-HIV integration literature and policy documents, and interviews with an expert panel, we proposed and applied a PrEP-FP integration framework that delineates the systems issues that must be addressed for effective integration, particularly for AGYW.

## Materials and Methods

This programmatic analysis involved three steps. First, we conducted a desk review of published reviews and policy documents related to HIV-FP service delivery integration and oral PrEP introduction to inform the development of a PrEP-FP integration framework. Next, we conducted interviews with 30 experts at the global level and across East and southern Africa to further refine the framework. Finally, we applied the framework to seven countries with high levels of HIV burden and at varying stages of PrEP rollout—Kenya, Lesotho, Malawi, South Africa, Uganda, Zambia, and Zimbabwe—identifying barriers to and enablers of PrEP-FP integration along the framework.

The desk review included five global review articles purposively selected to provide context on the current state of evidence regarding HIV and FP integration (15–18, 24). The desk review for Lesotho, Malawi, South Africa, Uganda, and Zambia included 61 policy and program sources (11–14 per country) related to country plans and policies, integration information (site audits and reports), and demographic information (25). For Kenya and Zimbabwe, information was sourced from the HIV Prevention Market Manager reports on integration of HIV prevention and SRH services in each country, which were based on policy reviews, expert interviews, facility assessments, and youth consultations (26, 27).

The interviews were conducted with a convenience sample of 30 experts, including six of the authors, who were engaged in programmatic and technical support for PrEP and/or FP implementation and represented both global and country perspectives. They included seventeen national implementers (3 from Kenya, 2 from Lesotho, 2 from Malawi, 4 from South Africa, 1 from Uganda, 2 from Zambia, 3 from Zimbabwe), one national policymaker (from Uganda), seven global implementers, and five staff from the United States Agency for International Development (USAID) (25). Interviews were conducted virtually with individuals or in small groups of two to three persons. The interviews were recorded with each participant's verbal permission, and notes were transcribed into a Word document. Prior to the interviews, the draft PrEP-FP integration framework was shared with participants. During the interviews, participants were asked to provide input on the framework components, as well as country-specific feedback on activities related to PrEP-FP integration. Interview discussion themes were structured around five health system domains: plans and policies, resource management, service delivery, PrEP use, and monitoring and reporting.

Based on the desk review and expert interviews, the components of the PrEP-FP integration framework were refined and a general assessment of progress toward integration was mapped across the seven countries, including identifying common barriers to and enablers of PrEP-FP integration.

## Results

**Figure 1:**
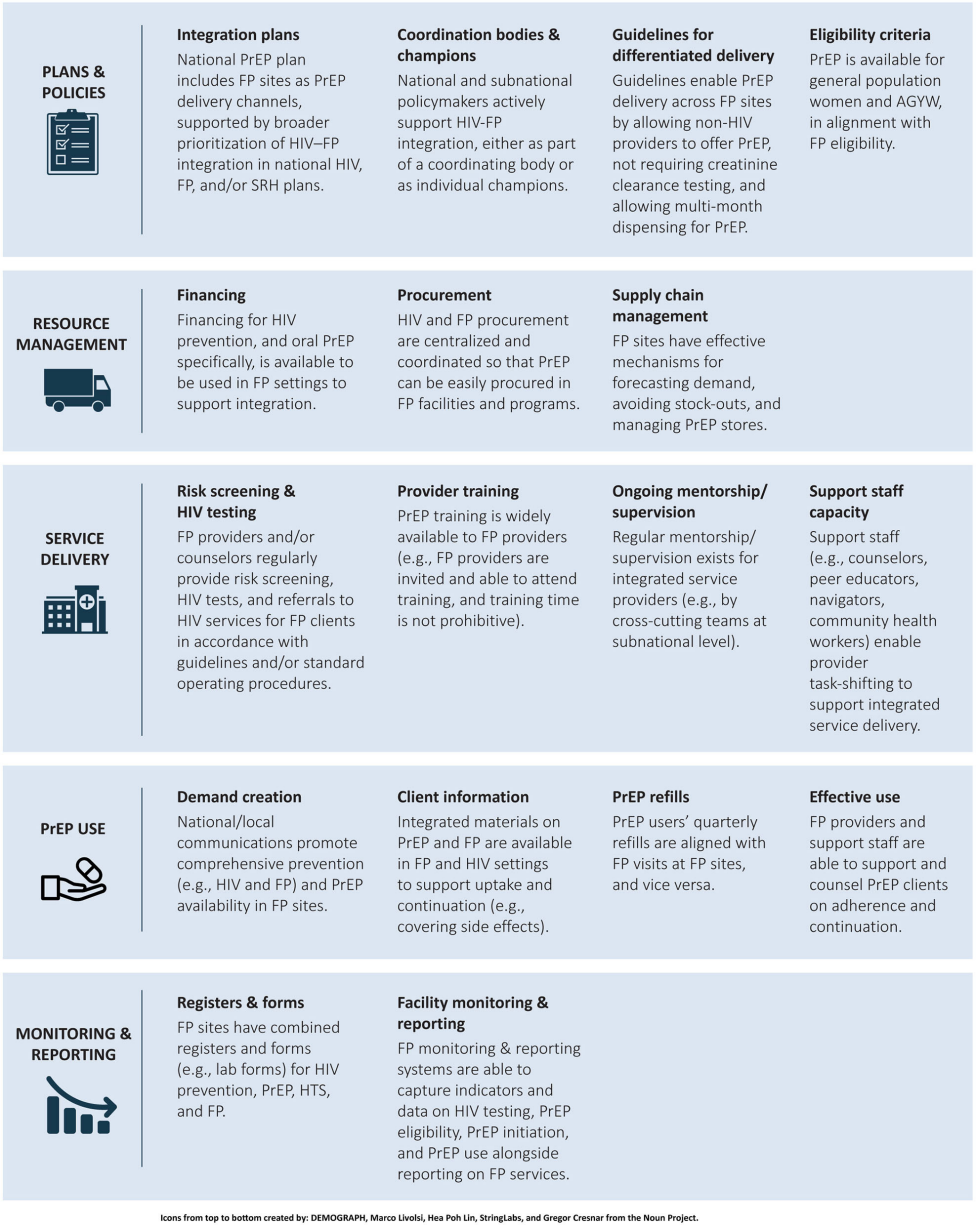
PrEP–Family planning integration framework. PrEP, pre-exposure prophylaxis; FP, family planning; SRH, sexual and reproductive health; AGYW, adolescent girls and young women; HTS, HIV testing services.

The analysis identified 17 essential elements to support PrEP-FP integration across five major health system domains: plans and policies, resource management, service delivery, PrEP use, and monitoring and reporting. These elements formed the foundation of the PrEP-FP integration framework (see Figure 1) (25).

**Figure 2:**
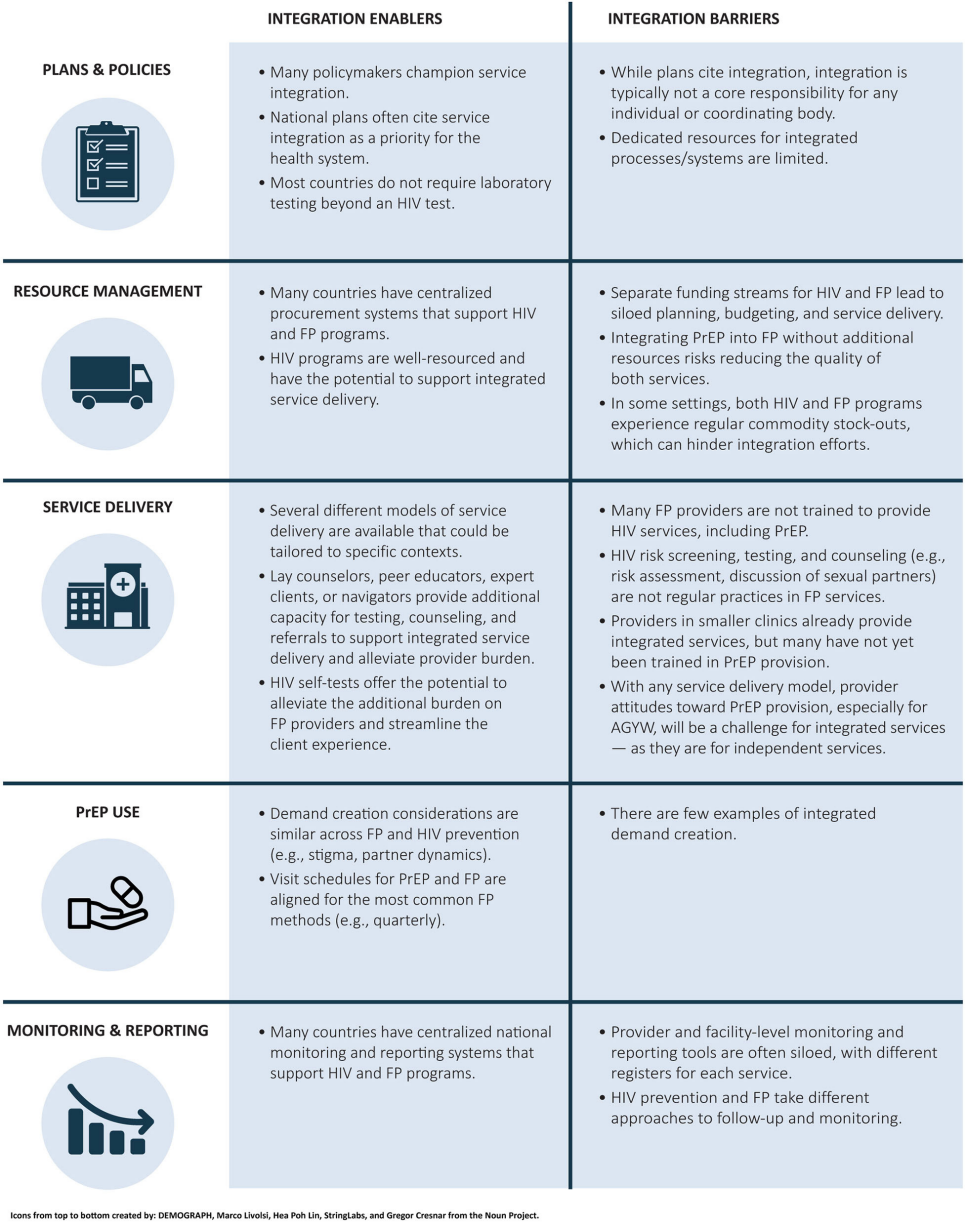
Summary of key findings: consistent patterns across seven countries. FP, family planning; PrEP, pre-exposure prophylaxis; AGYW, adolescent girls and young women.

During the assessment based on this framework, consistent patterns emerged across the seven countries that highlighted barriers to and enablers of integration (see Figure 2) (25). The key findings for each category are presented in sections Plans and policies, Resource management, Service delivery, PrEP Use, and Monitoring and reporting.

### Plans and Policies

Leadership and dedicated human and financial resources are essential to support PrEP-FP integration. Across the countries included in this analysis, policymakers and donors consistently supported integrated service delivery, and every country had national policy documents promoting the delivery of integrated health services, including HIV prevention and FP services. Several countries had also introduced national initiatives specifically focused on multisectoral approaches to AGYW well-being, such as the She Conquers (28) campaign in South Africa, which aimed to align services to reduce HIV incidence, unplanned pregnancy, and incidence of intimate partner violence, as well as school dropouts and unemployment. In practice, however, structures for coordinating services typically remained siloed. Across all the countries, HIV prevention, including PrEP, and FP were managed by different Ministry of Health departments and separate national-level technical working groups. A stakeholder in Malawi noted, “Integration is being discussed at a national level, but who really champions integration? A missing link is that there is no unit in charge of integration.”

Few countries have tasked an individual or department with managing integration at the national and subnational levels, although both are critical to support implementation of integrated service delivery. For example, in Zimbabwe, a national HIV-SRH Integration Officer sits within the Family and Child Health Department of the Ministry of Health and Child Care (MoHCC), but provincial-level responsibilities are split between separate SRH and HIV focal persons. One potential exception is Kenya, where a renewed push to integrate HIV prevention and FP services is being led by a national subcommittee formed in July 2020 with joint membership from the National AIDS and STIs Control Programme (NASCOP) and the Department of Reproductive and Maternal Health, with an aim to replicate the structure at subnational levels in future years.

More specific policies can also enable or impede service integration. For example, guidelines that support differentiated service delivery and task-shifting are often different across PrEP and FP services. In contrast to a concentrated effort to ensure most FP services can be delivered by a range of providers, including community health workers, and across diverse channels, including community-based programs and pharmacies, PrEP is largely delivered via clinicians, including clinical officers, doctors, or nurses specifically trained in HIV care. Changes in policies on task shifting and expansion of the provider cadres that can deliver PrEP may be needed to enable and support PrEP-FP integration. Similarly, other policies on PrEP provision, including requirements for laboratory tests, multi-month dispensing, and age restrictions, also need to be considered to support alignment with provision of FP services. As noted by a stakeholder in Zambia, “Our public health FP facilities are very crowded. We need multi-month dispensing for both PrEP and FP and to think about what a community health worker can do. Without that, I don't know how we will manage it.”

### Resource Management

PrEP and FP funding for commodity procurement, distribution, and management also need to be considered to support integrated services. One of the largest challenges to effective PrEP-FP integration is a difference in financing for the two services. PrEP has largely been donor-funded, with extensive dedicated resources to support PrEP provider training, service delivery, and monitoring. FP services, on the other hand, have transitioned to being funded with a combination of domestic and dedicated donor funds. Separate funding streams often contribute to silos in planning, budgeting, and delivering PrEP and FP services. For example, a South Africa implementing partner noted the challenge that donors can pose to service integration: “Funding can be a real challenge. Within our work on PrEP, we were trying to also improve access to FP for AGYW, because that is such a big gap. But every time we included FP elements, we had to justify how it helped to meet the 90-90-90 goals. It was an uphill battle.”

In terms of resource management, most of the countries included in this analysis have centralized public procurement systems that manage both PrEP and FP commodity procurement. However, siloed funding results in parallel systems for commodity procurement and distribution, creating a barrier to integration. An implementing partner from Lesotho highlighted the challenges that siloed resource management systems can have throughout the supply chain: “Integration would be ideal, but commodities are our biggest challenge. In our area, we are responsible for PrEP implementation, and another NGO is responsible for FP implementation. So, the district manager will not allow us to get FP commodities. For now, we have trained providers to deliver both PrEP and FP, but we cannot get FP commodities. It requires coordination across multiple units within the Ministry of Health, and those silos then flow down to the service point.” While this example focuses on the integration of FP into PrEP service delivery, it nonetheless offers insight into supply chain challenges faced when integrating HIV prevention and FP services.

### Service Delivery

**Figure 3:**
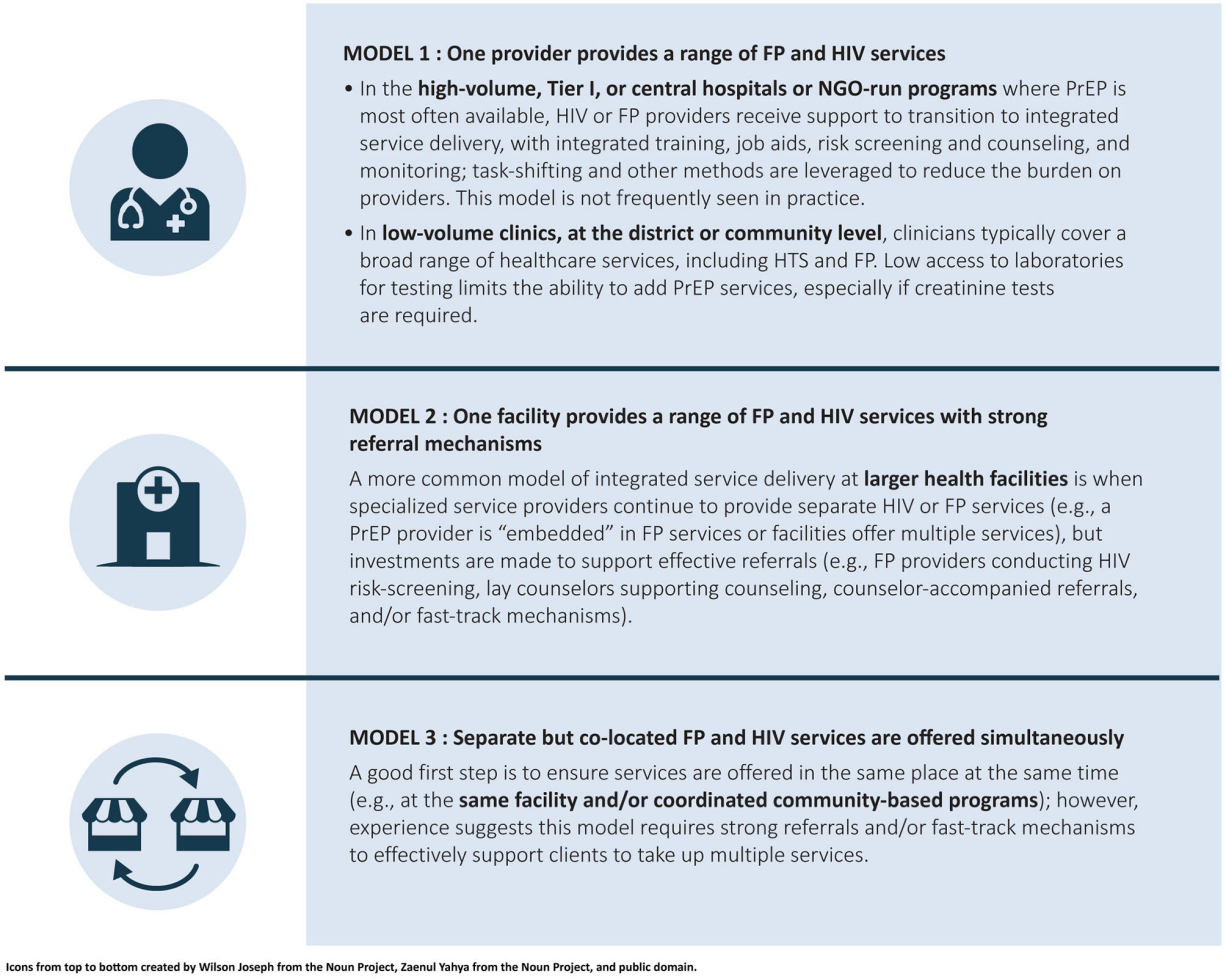
PrEP-FP integration models. PrEP, pre-exposure prophylaxis; NGO, nongovernmental organization; FP, family planning; HTS, HIV testing services.

There are several models for PrEP-FP integration, each of which has benefits and challenges (see Figure 3) (25). One model is to equip FP providers to offer both FP and PrEP services in a single visit. This is the most streamlined model from the end-user perspective, because it requires only a single visit to a provider to access a complete range of services. In this model, however, FP providers must provide HIV testing services (HTS), carry out HIV risk assessment and PrEP baseline assessment, and provide PrEP counseling and services. To do so effectively, FP providers must have the necessary training, mentorship, and supervision on HIV prevention and PrEP specifically. HTS and PrEP services would also need to be included in job descriptions, standard operating procedures, and job aids for FP providers. In the high-volume facilities where PrEP is most likely to be available, this model carries a high risk of disrupting FP service delivery because it requires more time from FP providers, diverting their time from provision of contraceptive services. In low-volume or rural settings, where providers already provide a range of integrated services, including FP and HIV prevention, this model could be effective. However, it was rarely employed to support integrated PrEP-FP service delivery in the seven countries.

Another model is to offer both FP and PrEP services in a single facility or community-based setting, with systems of referrals between different providers and service delivery points. In this model, FP providers make referrals to PrEP providers for those who need both services. Referrals can be made early in the process—for example, a referral for HTS—or later, for example, with an FP provider conducting HTS and HIV risk assessment, and then making a referral to a PrEP provider for PrEP counseling, eligibility determination, commodity provision, records management, and follow-up care. One way many programs were operationalizing this model was to have a dedicated PrEP provider available to screen, counsel, and support PrEP clients in FP settings. This approach supports integrated service delivery for end users without requiring as much integration of back-end systems, including funding and monitoring and reporting. Some programs employing this second model were also using referrals with a “fast-track option” that allows clients to avoid waiting in multiple queues to help improve end-users' experiences.

While co-locating services offered by different providers and providing structured referrals is not as streamlined for end users as the first model, it is another way of operationalizing integration at the facility and provider levels. Elements of this second model are already in place in many of the countries included in this analysis: HTS is widely available in FP settings and HIV risk assessment is part of standard practice for FP visits in more than half of the countries. As such, this is the most commonly adopted integration model across countries, with a particular focus on reaching HIV-negative AGYW. As a stakeholder in Uganda noted, “Most FP providers have basic training on HCT [HTS], but it does not include PrEP yet. With limited resources, we are rolling out PrEP training in FP settings specifically in the facilities or geographies where we will get maximum benefit, with a focus on AGYW.”

A third model is to offer both FP and PrEP services in the same facility or setting at the same time, but without a structured referral system. This approach could include, for example, coordinating dates and locations for community-based PrEP and FP services. Although this model might be the easiest to implement, it offers less efficiency from the end-user perspective. For example, a collaboration between two implementing partners to offer community-based PrEP and FP services in Lesotho demonstrates some of the challenges: “We are trying to collaborate to co-locate services so that clients can move from tent to tent, but we have not seen good results because clients do not want to go and join another queue and see a new face.”

Some integration models may require significant changes at the point of service delivery, as is often seen in programs led by implementing partners or specific adolescent-friendly services that are designed to offer a “one-stop shop” approach. In these settings, programs and facilities were also testing approaches to minimizing burdens on providers and disruption of existing services, including shifting some tasks to cadres of lay healthcare workers or using self-administered risk assessments and HIV tests. As a stakeholder in Kenya noted, “FP services typically quickly dispense boxes of contraceptive pills. Incorporating something that takes 20 to 30 minutes per client is a lot. Having some way to do screening before getting to the provider helps. We are also looking to implement HIV self-testing, so that women can take an HIV test as they are waiting for FP services to reduce health personnel bottlenecks.”

### PrEP Use

Integrated demand creation for both PrEP and FP is critical, to build awareness of PrEP as an intervention and a component of an integrated service package, and to foster awareness of its availability in FP settings. Integrating demand creation efforts can also be cost-effective and mutually beneficial for PrEP and FP goals, because both services reach similar populations and seek to expand access to and use of preventive health services, often with a lens to strengthening women's empowerment and health autonomy. South Africa, for example, has established the B-Wise (29) platform focused broadly on AGYW health, which could serve as a platform for both FP and PrEP. As a stakeholder from South Africa shared, “On the ‘B-Wise' site, people can come with questions and talk to a chatbot or a helpline, and then get linked to a healthcare provider. The whole approach is to promote self-care around different needs. Not just a pregnancy test, but also STI screening and HIV prevention, so that young people can understand their comprehensive needs.” Although all the countries included in this analysis had some demand creation investments for PrEP or FP, integrated approaches to demand creation were rare.

Several projects offering integrated PrEP-FP service delivery allow for multi-month dispensing of PrEP so users can align clinic visit schedules for quarterly HIV tests and PrEP and short-acting FP refills. This approach is particularly helpful for those who use contraceptive pills or injections but is less useful for those who use long-acting methods, such as an implant or intrauterine device. Across the seven countries, more than 50% of women and girls of reproductive age used the contraceptive pill or injection; however, to fully support women's needs for integrated services, PrEP-FP integration models must develop an efficient approach to follow-up for women using long-acting or permanent contraception.

### Monitoring and Reporting

Differences in the nature of PrEP and FP service delivery are also reflected in monitoring and reporting for these services. None of the seven countries had a fully integrated monitoring and reporting system for PrEP and FP services. At the facility level, this means that providers offering integrated services must complete multiple, separate registers during a single visit to account for the different services provided. As a stakeholder in Zimbabwe noted, “The registers should be integrated so that providers can complete one register across all services. As long as they are separate, healthcare providers will continue to see different activities [PrEP provision] as add-ons and not core to their work.” Some programs did record a limited number of indicators on integrated services, such as tracking FP users who received HTS at their last visit.

This challenge is amplified within national health information systems, where PrEP and FP services are monitored and assessed very differently. PrEP programs aim to track clients through individual client records, and follow-up is managed to achieve quantitative site-level targets for initiation and continuation. Due to the emphasis on the voluntary nature of FP services, contraceptive clients are rarely monitored, and programs use aggregate metrics to track overall numbers of FP users and contraceptives dispensed rather than progress toward site-level targets. As a global expert noted, “There is generally little data or recordkeeping for FP services. FP clients may have a services card and FP sites have registers, but there are no individual client files. On the PrEP side, there is a lot of paperwork. How can these be accommodated in FP services? In FP rooms? Who will be responsible? PrEP may provide an opportunity to improve monitoring of integrated services, but it will require a lot of support.” Stakeholders noted that emerging investments in individual-level electronic medical records will help support monitoring and recordkeeping across services in the future.

## Discussion

Integrated service delivery is a complex, multifaceted undertaking that requires a system-wide approach (15). This paper describes a PrEP-FP integration framework that delineates enablers of and barriers to PrEP integration with FP services across five domains. When the framework was applied as a matrix across seven countries in East and southern Africa, patterns emerged revealing critical gaps that may hinder progress toward PrEP-FP integration. For example, many of the countries had established policy frameworks to support integrated service delivery, but areas such as coordination, resource management, provider training, demand creation, and monitoring and reporting had yet to be addressed. While support for PrEP-FP integration was strong, implementation examples were limited to a few programs, many of which were funded through research programs or non-governmental organizations (NGOs) rather than the public sector. Given the relative newness of PrEP, few studies evaluating the integration of oral PrEP with FP services in Africa have been published (21, 30). Further research is needed to determine whether PrEP-FP integration can help overcome barriers to PrEP uptake and continuation, and whether integrated delivery impacts the quality of care for FP and HIV prevention services.

### Plans and Policies

Despite PrEP being a relatively new service, many countries are moving forward with national policies and guidelines to support PrEP-FP integration, building on broader efforts to promote HIV-SRH integration across services and populations. However, as highlighted in the PrEP-FP integration framework, efforts are still needed at the policy level to ensure coordination and collaboration between HIV/PrEP and SRH/FP departments, along with identifying which national body or bodies will be responsible for PrEP-FP integration. Given the decentralization of services to the district level in most countries, and even to the facility level in some countries, it is critical that any integration efforts reach the subnational level. Without the constructive engagement of local government officials, health providers, and community stakeholders, it is unlikely integration will succeed.

### Resource Management

Resource management is a more challenging domain because investments are needed for commodities and integration of supply chains, training, demand creation, and monitoring and evaluation systems. HIV programs are generally better funded than SRH/FP programs, and government and donor funding for these programs is often siloed; hence, coordination across donors, ministries of health, and subnational actors is essential to support PrEP-FP integration. Recent experience with the integration of HIV testing into FP services suggests that integration of HIV services with FP is feasible, and governments should consider building on these efforts to support PrEP-FP integration (18).

### Service Delivery

Similar to broader SRH-HIV integration efforts, PrEP-FP integration will likely rely on a variety of integration models ranging from one-stop shops to enhanced referrals (17). Determining which model fits best in a given context will require the thoughtful engagement of policymakers, service providers, program managers, and clients, together with consideration of other FP integration priorities beyond PrEP (e.g., STI screening) and population-specific needs. Most countries in our analysis had made progress integrating HIV testing into FP services, which provides a natural link to then provide counseling and offer PrEP to those who test negative. Previous efforts at SRH-HIV integration found that providers miss opportunities to integrate care and programs face challenges to maintaining quality of care with integrated service delivery (24). As identified in the PrEP-FP integration framework, critical areas that need to be addressed to overcome these pitfalls include provider training, ongoing mentorship, and capacity building for ancillary staff (i.e., lay counselors, peer navigators, and community health workers). Care must be taken to ensure that the large majority of women of reproductive age who seek FP services—who may not need PrEP services—are not disenfranchised by the inclusion of the new service or subjected to a lower quality of care due to increases in client volume or provider workloads.

### PrEP Use

To support PrEP use for those accessing PrEP in FP settings, engagement with clients must be integrated across the client journey—starting with demand creation efforts and continuing through to follow-up care. While integrated messaging has the potential to be more cost-effective and mutually beneficial for both HIV prevention and FP goals, the challenge remains that demand creation efforts are not well-funded or sustained in either health area. Nevertheless, governments can influence a more integrated effort, as evidenced in the South Africa B-Wise example described above. Additional efforts are needed to align PrEP refill schedules with FP services and to integrate outreach (e.g., by peer ambassadors) and counseling to support informed decision making and help reduce discontinuation of both PrEP and contraception.

### Monitoring and Reporting

The difference in intensity of data reporting requirements (e.g., PEPFAR requirements for PrEP compared to demographic and health management information system requirements for FP) and the common use of separate registers for different services makes it difficult to integrate monitoring and reporting. Initially, the PrEP monitoring approach in sub-Saharan Africa mirrored that of HIV treatment services rather than those of comparable prevention models such as FP. Shifting this mindset will require intentional effort by governments and donors to consider alternative requirements for and methods of PrEP reporting. At the same time, many unanswered questions about PrEP remain, particularly as new products come on the market and as PrEP-FP integration efforts are nascent. Early PrEP-FP integration should be assessed to inform ongoing efforts, and investments will be necessary to support the additional data collection needed to monitor PrEP-FP delivery. A recent review highlights the potential of electronic health information systems to facilitate coordination across services, which may be an effective solution for monitoring and reporting of integrated health service delivery (31).

## Limitations

The PrEP-FP framework described in this paper is limited by the fact that it was a high-level programmatic effort, with a focused desk review and expert interviews in a select group of countries in East and southern Africa. The experts consulted were a small, convenience sample including varied representation across seven countries, only one national policymaker, no providers, and six of the authors, which limits the generalizability of this programmatic effort. The framework focuses specifically on integrating PrEP into FP services and does not examine barriers and enablers associated with integrating PrEP into other areas, such as maternal health and child health services. It also does not address whether PrEP-FP integration can increase PrEP uptake. The framework was developed with a lens on general-population women and AGYW; specific integration needs for key populations, such as female sex workers, deserve further attention. Finally, while the experts interviewed reflected on a wide range of service delivery approaches, the primary emphasis was on facility-based rather than community-based delivery, given that most PrEP is currently provided through health facilities. However, community-based delivery of PrEP is expanding, and further exploration of the enablers of and barriers to PrEP-FP integration in these settings is warranted.

## Conclusions

While there is broad interest in integrating PrEP into FP services, there are key differences between these services that manifest not only at the site of service provision but also throughout different elements of the health system. The PrEP-FP integration framework we developed highlights the multiple system factors that need to be in place to facilitate integrated service delivery. The HIV prevention field is expanding rapidly, with new products on the near horizon and the potential for at least one antiretroviral-contraceptive multipurpose technology (MPT) to enter the market in the next 5 years. Addressing PrEP-FP integration now will facilitate the introduction of MPT products in the future. At the same time, focusing these efforts on achieving a “win-win” for both FP and PrEP (HIV) is a key consideration in moving integration beyond the conceptual level to full implementation. Evidence demonstrating that client use of both services increased, client satisfaction improved, and efficient and feasible approaches to service management and provision were identified would make the path to wider-scale adoption of PrEP-FP integration more likely.

The full PrEP-FP integration framework and matrix, publicly available on the PrEPWatch website, is a programmatic tool that may be adapted to different settings, be these country-specific or other types of HIV-SRH integration efforts (25). Our hope is that the framework can help policymakers and program managers take a comprehensive, systems approach to integrated PrEP-FP delivery, assess opportunities for progress, and anticipate key challenges in their settings.

## Data Availability Statement

The datasets presented in this study can be found in online repositories. The names of the repository/repositories and accession number(s) can be found below: https://www.prepwatch.org/wp-content/uploads/2020/07/OPTIONS_PrEP_FP_IntegrationAnalysis.pdf.

## Author Contributions

NB conceptualized the framework, conducted the desk review, interviews and analysis, contributed to writing the manuscript, and interpretation of the findings. KT, RW, RR, SM, IM, JR, and JM contributed to the adaptation of the framework, the writing of the manuscript, and interpretation of the findings. JS contributed to the writing of the manuscript and interpretation of the findings. All authors contributed to the article and approved the submitted version.

## Conflict of Interest

The authors declare that the research was conducted in the absence of any commercial or financial relationships that could be construed as a potential conflict of interest.
